# Reduction of Grain Boundary Resistance of La_0.5_Li_0.5_TiO_3_ by the Addition of Organic Polymers

**DOI:** 10.3390/nano11010061

**Published:** 2020-12-29

**Authors:** Iker Boyano, Aroa R. Mainar, J. Alberto Blázquez, Andriy Kvasha, Miguel Bengoechea, Iratxe de Meatza, Susana García-Martín, Alejandro Varez, Jesus Sanz, Flaviano García-Alvarado

**Affiliations:** 1 CIDETEC, Basque Research and Technology Alliance (BRTA), P Miramón 196, 20014 Donostia-San Sebastián, Spain; aramos@cidetec.es (A.R.M.); ablazquez@cidetec.es (J.A.B.); akvasha@cidetec.es (A.K.); mbengoechea@cidetec.es (M.B.); imeatza@cidetec.es (I.d.M.); 2 Inorganic Chemistry Department, Facultad de Ciencias Químicas, Universidad Complutense, 28040 Madrid, Spain; sgmartin@quim.ucm.es; 3 Department of Materials Science and Engineering, Universidad Carlos III de Madrid, Avda. de la Universidad 3, Leganés, 28911 Madrid, Spain; alvar@ing.uc3m.es; 4 Department of Ionic Solids, Instituto de Ciencia de Materiales (CSIC) Sor Juana Inés de la Cruz 3, Cantoblanco, 28049 Madrid, Spain; jsanz@icmm.csic.es; 5 Chemistry and Biochemistry Department, Facultad de Farmacia, Universidad San Pablo-CEU, CEU Universities, Urbanización Montepríncipe, Boadilla del Monte, 28668 Madrid, Spain; flaga@ceu.es

**Keywords:** lithium lanthanum titanium oxide (LLTO), grain boundary resistance, solid ceramic-polymer composite electrolyte, lithium ion conductivity

## Abstract

The organic solvents that are widely used as electrolytes in lithium ion batteries present safety challenges due to their volatile and flammable nature. The replacement of liquid organic electrolytes by non-volatile and intrinsically safe ceramic solid electrolytes is an effective approach to address the safety issue. However, the high total resistance (bulk and grain boundary) of such compounds, especially at low temperatures, makes those solid electrolyte systems unpractical for many applications where high power and low temperature performance are required. The addition of small quantities of a polymer is an efficient and low cost approach to reduce the grain boundary resistance of inorganic solid electrolytes. Therefore, in this work, we study the ionic conductivity of different composites based on non-sintered lithium lanthanum titanium oxide (La_0.5_Li_0.5_TiO_3_) as inorganic ceramic material and organic polymers with different characteristics, added in low percentage (<15 wt.%). The proposed cheap composite solid electrolytes double the ionic conductivity of the less cost-effective sintered La_0.5_Li_0.5_TiO_3_.

## 1. Introduction

Conventional lithium ion batteries have been widely used for cellular phones and personal computers because of their high operational voltage and high energy density. Moreover, electrical vehicles (EV) are also presently a target for lithium ion battery producers. However, due to the wide use of a flammable organic liquid electrolyte, they lack inherent safety, which is one of the main urgent concerns to enable further implementation of next-generation high-energy batteries for electrical propulsion.

Room temperature ionic liquids (RTILs) have been increasingly recognized as promising electrolyte candidates to replace the traditional and volatile liquid organic electrolyte systems [1,2]. The main advantage of RTILs against organic solvents is the intrinsic safety and nonflammability; the negligible vapor pressure; high chemical, electrochemical, and thermal stability. However, when the performance of RTIL-based electrolyte systems is compared with that of systems containing conventional electrolytes, further improvement is still necessary. A disadvantage for the RTILs is the required high purity, since impurities (especially, halogens Br^−^, Cl^−^), even in trace amounts, significantly affect their electrochemical properties and, as a result, the compatibility with conventional battery electrode materials (Li metal, NMC cathode, etc.). Additionally, their synthesis is far from environmentally friendly, since it generally requires anion exchange. These drawbacks, together with the high price of common ILs, hamper their industrial emergence, and new concepts are now strongly needed in order to utilize these systems in a more rational way.

Solid polymer electrolytes (SPEs) also offer numerous advantages over current liquid organic electrolytes [3]. This kind of electrolyte enhances the energy, operating temperature range, shelf life, and reversibility. SPEs are ionic conductive solids based on macromolecules containing heteroatoms that allow the dissolution of one or several salts and enable their diffusion under an applied electric field. Furthermore, SPEs offer several advantages over conventional liquid electrolytes, such as a good mechanical strength with some ability to deform, easy handling which allows for the fabrication of self-standing films and the elimination of the problem of battery electrolyte leakage. However, the principal problem associated with this type of electrolytes is their low ionic conductivity at ambient temperature and limited electrooxidation stability.

In this context, more recently, inorganic solid electrolytes have drawn much attention thanks to their intrinsic advantages in terms of electrochemical and thermal stability, together with a very high ionic mobility at ambient temperature [4]. Nevertheless, solid inorganic electrolytes present disadvantages that have to be overcome, such as the need for ceramic (high-temperature) processing, a high electrode-electrolyte interface resistance, or the lower ionic conductivity in comparison to the liquid systems [4].

Among the solid electrolytes reported so far, several materials have shown to be promising candidates for practical application in lithium ion batteries, such as sulphide glasses [5,6,7,8], sulphide crystalline materials [9,10,11], garnet-type Li_7_La_3_Zr_2_O12 (LLZO) [12], NASICON-type oxides [5], or germanium-based Li_1.5_Al_0.5_Ge_1.5_(PO_4_)_3_ (AGP) [4]. Another interesting material is the perovskite lithium lanthanum titanate (LLTO) (stoichiometry La_2/3-x_Li_3x_TiO_3_), which presents higher lithium ion bulk conductivity (10^−3^ S cm^−1^) compared to other oxide-type solid electrolytes [13,14,15].

The grain boundary resistance, being two orders of magnitude higher than the bulk resistance, limits the total conductivity of the polycrystalline LLTO to the typical range of 10^−5^–10^−4^ S cm^−1^ at 25 °C. This fact limits the use of these materials as solid electrolytes [16]. In this context, the ceramic microstructure, e.g., grain size and morphology, as well as the lithium content, plays a key role on the ionic conductivity of LLTO-based solid electrolytes [17].

In particular, some works have been focused on reducing the grain boundary effect to improve the overall conductivity of LLTO materials. So far, several approaches, for example the adjustment of the battery design [18], the optimization of the sintering process of LLTO ceramics [16], the substitution of A and B-sites by other atoms, and the introduction of a second phase into the LLTO [17], have been proposed to enhance the ionic conductivity.

Processing parameters such as sintering time and temperature significantly affect the total conductivity [17]. The solid electrolytes should be shaped into a thin layer with a large area in order to obtain low internal resistance and, thus, to yield a high current. Ceramic technology provides some non-expensive methods to form layers that, at the final step, have to be sintered (for example, tape casting), which increases the final price of the material. However, even though sintering-based processes may be suitable for preparing thin structures (<0.5 mm thick), the all-ceramic electrolyte layers are inherently brittle and thereby difficult to manufacture [19]. In addition, the high sintering temperature and long treatment time in ceramic processing could lead to a serious loss of lithium, with a deleterious effect on lithium ionic conductivity [18].

Another way to reduce the grain boundaries of the solid electrolytes and to increase the final conductivity is to prepare a composite material by the addition of a polymer to an inorganic material [20]. The polymer influences the compaction process of the inorganic sample by filling the space between particles, which improves the contact of the electrode-electrolyte interfaces and the grain boundary contact. The approach would enhance the conductivity and the battery performance. Another advantage of using a composite electrolyte is that the mechanical properties and flexibility can be preserved [21].

**Figure 1:**
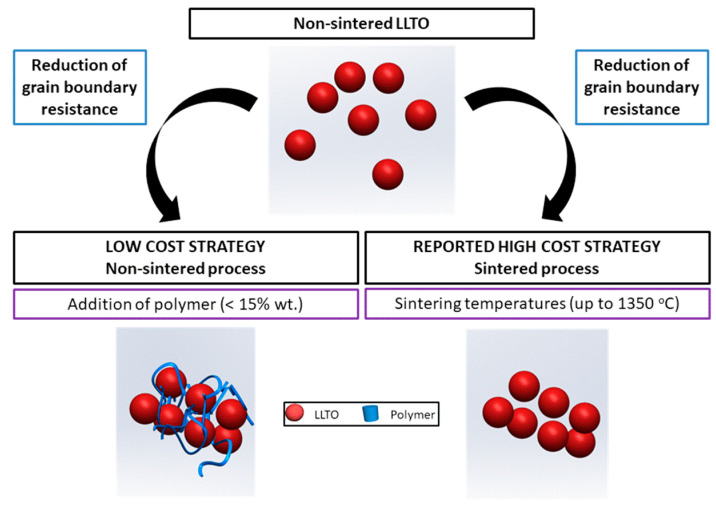
The strategy for a low-cost increased conductivity solid ceramic electrolyte followed in this work.

In most studies of ceramic composites, the polymer is the main component, and only a small quantity of inorganic material is added [4]. In this work, we investigated the opposite, the addition of small quantities of polymer as a low-cost process to reduce the grain boundary resistance of a ceramic solid electrolyte in order to reach an ionic conductivity similar to that of the high-cost sintered ceramic, LLTO in the case herein presented (Figure 1).

We study the effect of the addition of small quantities of different organic polymers (2–15 wt.%) on the conductivity of a solid composite electrolyte based on the most promising La_0.5_Li_0.5_TiO_3_ reported by Inaguma [22] using impedance spectroscopy. The selection of these polymers has been based on the following requirements [23]:

1. Low crystallinity, because the ion conduction in the organic part of the composite usually occurs in the amorphous part of the polymer
2. High lithium ion transference number, to enhance the kinetics of the electrochemical process and, thus, the rate capability of the battery.

Several polymers, poly(ethylene glycol) (PEG), polyacrylonitrile (PAN), poly(ethylene oxide) (PEO) and PEO copolymers (PEO/EP and PEO/EM/AGE) [24,25,26,27] have been chosen based on those two requirements, that are not frequently fulfilled at the same time. Thus, we started with the very well-known and standard PEO and some others that present either a lower degree of crystallinity or higher conductivity than PEO (see Table 1). However, they also present disadvantages [28], that are listed in Table 1.

## 2. Materials and Methods

### 2.1. Synthesis of La_0.5_Li_0.5_TiO_3_ Powder

La_0.5_Li_0.5_TiO_3_ samples were prepared by a conventional solid-state reaction. The reagents used for this study were as follows: Li_2_CO_3_ 99% (Merck KGAA, Berlin, Germany), La_2_O_3_ 99.99% (Sigma-Aldrich, Berlin, Germany), and TiO_2_ 99% (Sigma-Aldrich, Germany). La_2_O_3_ was heated at 1000 °C and TiO_2_ at 700 °C prior to weighing. Stoichiometric amounts of these reagents were ground together in an agate mortar and heated at 800 °C for 4 h in order to eliminate CO_2_. The reground mixture was then cold-pressed at 150 MPa and heated at 1150 °C for 12 h. These powders were used to produce the composites.

To prepare sintered LLTO pellets, the powder was reground, pressed, and heated again at 1350 °C for 6 h. In order to avoid lithium losses, the heating rate used in all treatments was performed at 1 °C/min. Finally, the sample was quickly cooled from high temperature by immersion in liquid nitrogen.

### 2.2. Composite Preparation

**Figure 2:**
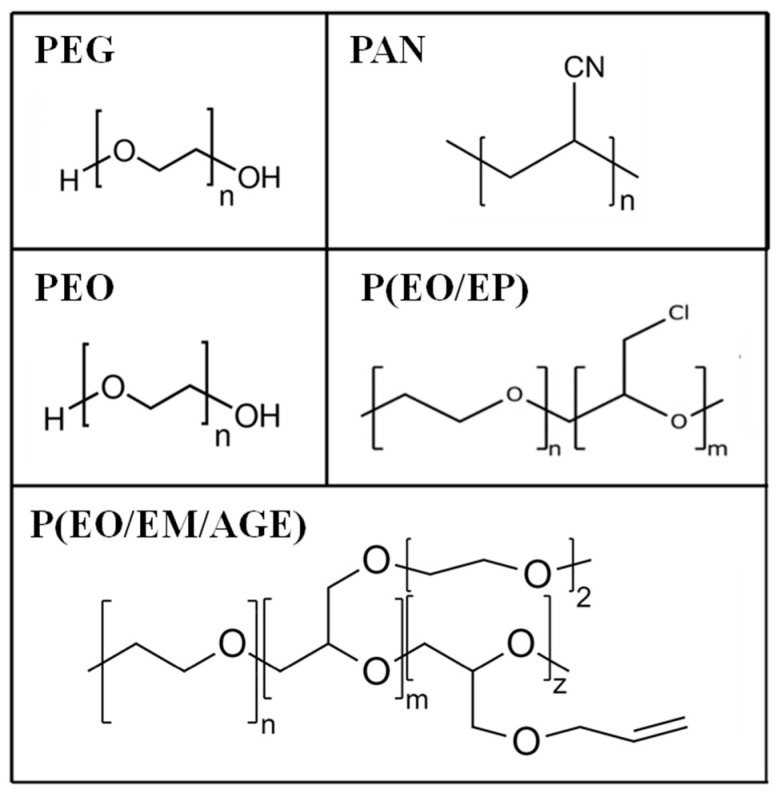
Chemical structures of poly(ethylene glycol) (PEG), polyacrylonitrile (PAN) poly(ethylene oxide) (PEO), and PEO based copolymers.

Polymers with different chemical structures (Figure 2) have been used to enhance the interface contact between the ceramic particles: poly(ethylene oxide) (PEO, M.W. 1.0 × 10^5^, Aldrich); poly(ethylene glycol) (PEG, Mw. 200, Fluka); PEO copolymers [poly(ethylene oxide-co-epichlorohydrin) P(EO/EP), Mw. 3.0 × 10^6^ and poly(ethylene oxide-co-2-(2-methoxyethoxy) ethyl glycidyl ether-co-allyl glycidyl ether) P(EO/EM/AGE), Mw. 1.5 × 10^6^, Dayso]; polyacrylonitrile (PAN, Mw. 1.0 × 10^5^, Aldrich).

Different amounts of polymer in weight (2%, 5%, 10%, and 15%) were added to the powdery inorganic material synthesized at 1150 °C, toluene was used as solvent for the PEG, PEO, and PEO copolymers, and DMSO was used as solvent for the PAN polymer. The mixture was stirred to form a homogeneous slurry which was poured onto a ceramic plate and allowed to evaporate the solvent slowly. All the samples have been prepared in dry conditions to avoid proton conductivity conditions.

### 2.3. Characterization Techniques

Impedance measurements of the composites were performed in dry conditions at room temperature. The reproducibility of the measurement has been followed by means of three parallel measurements. Additionally, sintered and non-sintered LLTO were prepared as pellets, by uniaxial pressing at 5 tons. Disk-shaped samples were sandwiched between stainless steel sheets as electrodes. The impedance measurements were carried out in the frequency range from 1 MHz to 0.1 Hz using a Solartron FRA1255B. The impedance data were fitted using the Zview 2.80 software. The ionic conductivity was calculated from Equation (1), where “d” and “A” are the thickness and area of the sample, respectively, and “R” is the measured resistance.

σ = (1/R) * (d/A)(1)

The impedance measurement shows the electrical behavior of the analyzed samples, which can be described by an equivalent electrical circuit. The resistivity and capacitance of each conduction process, associated to one equivalent circuit, can be quantified.

The scanning electron microscopy (SEM) images of the sintered and non-sintered LLTO, as well as the composite electrolytes, were taken at an accelerating voltage of 30 kV on a JEOL JSM5500LM microscope.

## 3. Results and Discussion

### 3.1. Characterization of the LLTO

#### 3.1.1. Morphology of the Sintered and Non-Sintered LLTO

**Figure 3:**
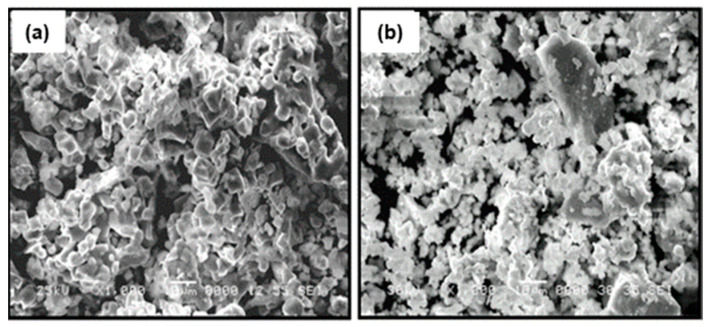
SEM micrographs of the surface of (**a**) sintered La_0.5_Li_0.5_TiO_3_ and (**b**) non-sintered La_0.5_Li_0.5_TiO_3_ pellets.

The particle morphology of the ceramic powder of La_0.5_Li_0.5_TiO_3_ used for the composites is shown in Figure 3a. As typical ceramic synthesis products, the particles are relatively large, mainly between <1 and 10 μm, with broad particle size dispersion due to the presence of even larger particles. The effect of a sintering process carried out at 1350 °C can be seen in Figure 3b. The sintering process at a temperature above the synthesis temperature increases particle size only slightly. In addition, the relative density is not very high. Advantageously, the sintering process has a significant positive effect in the grain boundary conductivity, as described below. The same effect is sought with the addition of organic polymers.

#### 3.1.2. Electrical Characterization of Sintered and Non-Sintered LLTO

**Figure 4:**
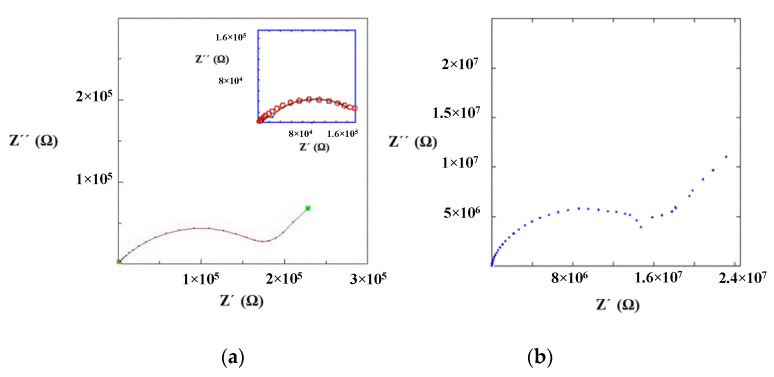
Impedance spectrum of (**a**) sintered and (**b**) non-sintered La_0.5_Li_0.5_TiO_3_ material at 25 °C.

**Figure 5:**
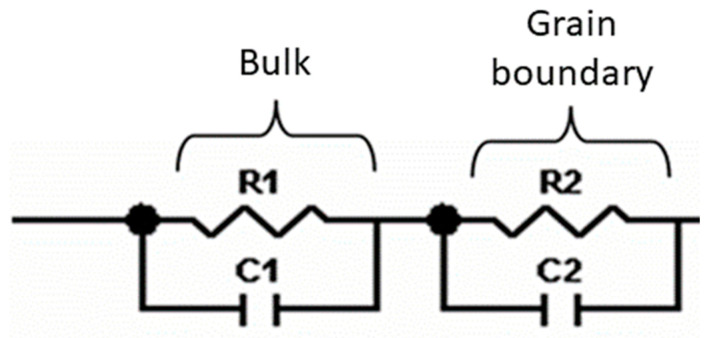
Equivalent circuit proposed to model the ionic conductivity process in La_0.5_Li_0.5_TiO_3_.

Impedance measurements were performed at 25 °C for La_0.5_Li_0.5_TiO_3_, sintered at 1350 °C, as well as a non-sintered pellet (Figure 4). The spectra consist of a small semicircle at high frequencies and a bigger second semicircle (inserted in Figure 4a). Finally, at very low frequencies the impedance shows a spike that is correlated to the blocking nature of the stainless steel electrode. The non-sintered inorganic material presents a wider semicircle due to its higher resistive nature. The region of interest to evaluate the grain boundary resistance corresponds to the first and second semicircles of the spectra. In this context, the equivalent circuit (Figure 5) used to describe the EIS spectra of a La_0.5_Li_0.5_TiO_3_ pellet is composed by two RC elements consisting in a resistance in parallel to a capacitor, related to the bulk and grain boundary contributions [29,30,31]. In the proposed model, universal capacitors [30] are used in order to take into account the capacitance dispersion. This phenomenon is generally attributed to distributed surface reactivity, surface inhomogeneity, roughness or fractal geometry, electrode porosity, and current and potential distributions associated with electrode geometry [31]. The universal capacitors are numerically expressed as Equation (2), where *B*(*i*ω) is the capacitance value and *a* expresses the distance of the capacitance from the ideal capacitor.

*C* = *B(iω)^a^* (2)

The fitting of the experimental data to this circuit model allows for the estimation of bulk and grain boundary resistances, which are listed in Table 2.

The maximum conductivities obtained for the bulk of sintered and non-sintered LLTO materials are 10^−3^ S cm^−1^. In both cases, the total ionic conductivity is dominated by the resistance of the grain boundary and, hence, the maximum conductivity can only be achieved upon the complete elimination of the inter-grain resistance.

The sintered LLTO presents a total ionic conductivity two orders of magnitude higher compared with the non-sintered one, due to the different nature of the interface between the particles as shown in the SEM images (Figure 3). In this context, the sintering of LLTO particles diminishes the resistance of the grain boundary, significantly increasing the ionic conductivity of the sintered LLTO solid electrolyte pellet.

#### 3.1.3. Characterization of Optimized Non-Sintered LLTO-Polymer Composites

**Figure 6:**
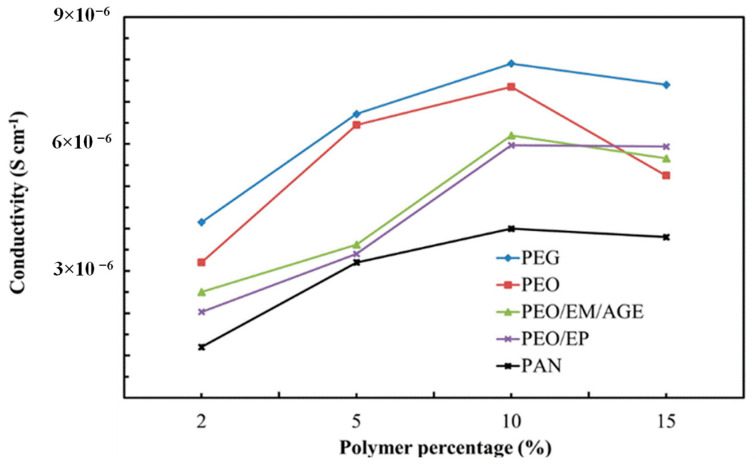
Ionic conductivity of composites with different polymers.

The amount of polymer has been firstly optimized to reach the highest conductivity of the ceramic-polymer composite electrolyte. We have used the same circuit model used for ceramic samples. Once the total conductivity of 2%, 5%, 10%, and 15% polymer-ceramic composites was estimated (Figure 6), we noted that the ionic conductivity increases with the polymer content, regardless of the particular polymer, reaching a maximum for 10 wt.%. This phenomenon is likely associated with the isolation of the inorganic particles. As a higher amount of polymer is added, the isolation of the LLTO particles is reduced and the ionic conductivity increases, since the ionic conductivity of polymer is higher than the conductivity in grain boundary of non-sintered LLTO grain boundaries. For values higher than 10 wt.%, the ionic conductivity decreases because conductivity is then dominated by the lower ionic conductivity of the polymer, now in excess and blocking percolation, when compared with the inorganic bulk.

The PEG-LLTO composite presents a higher total ionic conductivity, because, as shown in Table 1, PEG is the polymer with the highest ionic conductivity, followed by PEO. The PAN-ceramic composite exhibits the lowest ionic conductivity. Comparing the selected polymers, all of them, with the exception of PAN, present oxygen groups in their structure. Thus, it seems that the electronegativity of the oxygen group allows for better lithium ion conductivity [32].

Interestingly, the most 10%-polymer-LLTO composites show a total conductivity higher than the sintered LLTO. Thus, it is clearly demonstrated that these composites—and, in particular, the 10% PEG-LLTO composite—may provide a superior performance as lithium ion solid electrolyte in lithium ion batteries than LLTO.

#### 3.1.4. Characterization of the 10 wt.% Polymer-LLTO Composite

According to the results shown in Figure 6, 10 wt.% polymer-LLTO composites exhibit the highest ionic conductivity. Therefore, we have only characterized this particular composite solid electrolyte.

**Figure 7:**
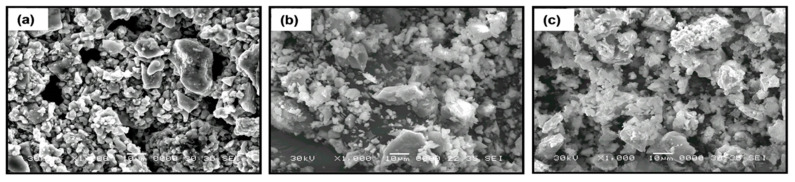
SEM micrographs of composite electrolytes formed by La_0.5_Li_0.5_TiO_3_ and 10 wt.% of polymer (**a**) PEG, (**b**) PEO, and (**c**) PAN.

The addition of 10 wt.% of polymer to non-sintered LLTO slightly modifies the distribution and the apparent surface morphology of the ceramic. The polymers appear as smaller globular particles adhered to the bigger and irregular ceramic grains (>10 µm). In the composites formed by PEG (Figure 7a), the polymer particles present smaller sizes and are more homogeneously distributed than in the composites with PEO (Figure 7b) and PAN (Figure 7c). This fact could be associated to the shorter PEG polymer chains (see Figure 2) and their lower viscosity, which enables their introduction into the free volume between the inorganic particles.

### 3.2. Electrical Characterization

#### Impedance Measurements and Ionic Conductivity of the 10% Polymer-LLTO

**Figure 8:**
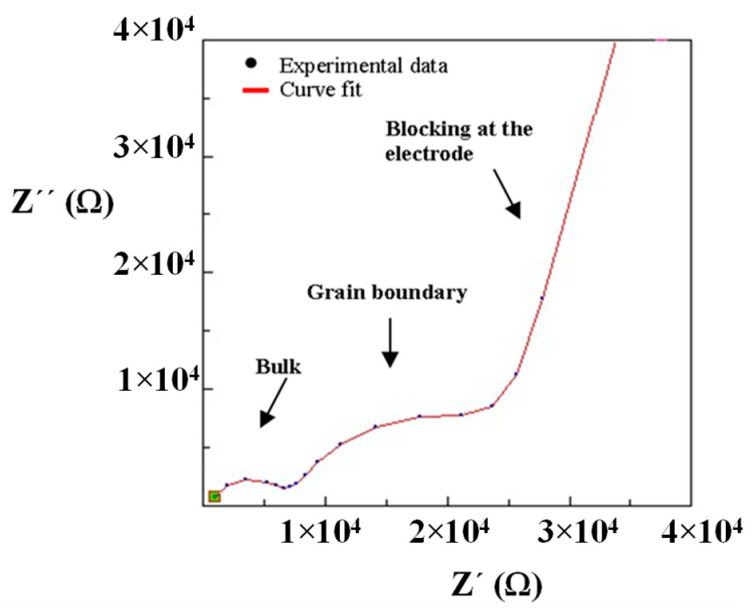
Impedance spectrum and curve fit of composite material (10% PEG/La_0.5_Li_0.5_TiO_3_) obtained at −20 °C.

In the composites, the bulk contribution cannot be clearly distinguished in the room temperature impedance spectra. Therefore, in order to calculate this process resistance, the impedance measurement has been performed at −20 °C for the composite 10% PEG-LLTO (Figure 8). At a lower temperature, the high frequency semicircle can be clearly seen and, hence, the high frequency intercept of the semicircle is chosen as the bulk resistance for the composite materials.

**Figure 9:**
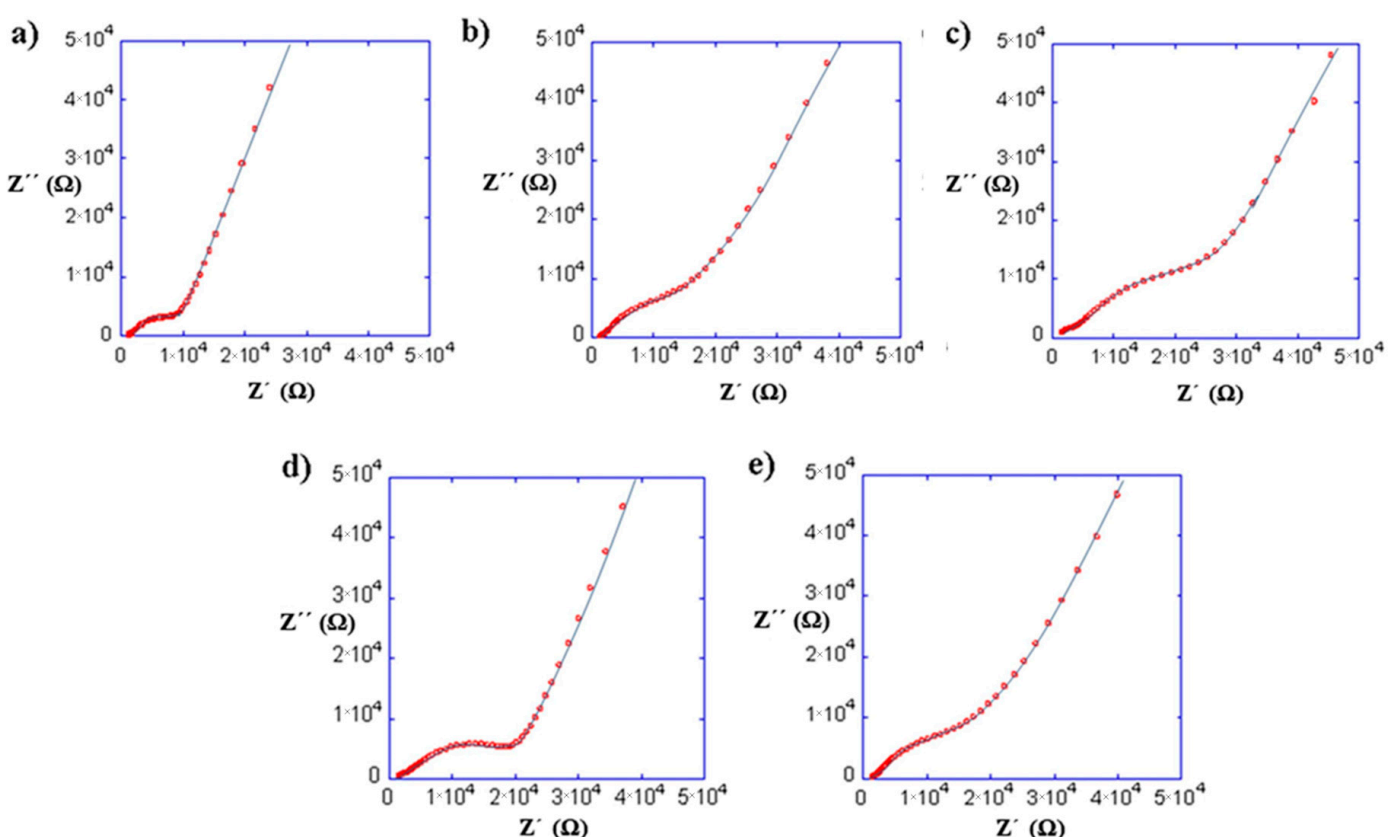
Impedance spectra at 25 °C of the composites with 10% of (**a**) PEO, (**b**) PEG, (**c**) PAN and PEO copolymers: (**d**) PEO/EP and (**e**) PEO/EM/AGE.

Figure 9 shows the impedance spectra at 25 °C of the composites prepared with (a) PEO, (b) PEG, (c) PAN and PEO copolymers: (d) PEO/EP and (e) PEO/EM/AGE. The curve fit has been obtained using the equivalent circuit shown in Figure 5 because the composites are mainly formed by the inorganic LLTO matrix. The bulk and interfacial process parameters of the composites with the total ionic conductivities are shown in Table 3.

The ionic conductivity associated to the bulk resistance for all the composites is around 10^−3^ mS cm^−1^. This fact confirms our assumption that the organic material only affects the grain boundary conductivity process. On the other hand, the capacity values of C2 are in agreement with the typical values assigned to the grain boundary or interface phenomena reported by Inaguma [22].

This study has been carried out without any salts as additional lithium ion source. The Li^+^ from the LLTO ceramic bulk was the only possible mobile ion, and was thus not likely to be a significant ion conductor in the polymer phase. Nevertheless, between the proposed polymers, different factors may have influence in the overall ionic conductivity of the composite, as the T_g_ of the polymer, the polymer distribution in the composite, or the presence of the oxyethylene group in the polymer chain which, will lead to a better distribution of polymer particles in the composite [32]. All these factors would explain the reduction of the grain boundary resistivity of the system.

Among the oxyethylene polymers, it was expected that the copolymers would reduce the grain boundary resistance further than PEO, due to their lower glass-transition temperature and, therefore, better mobility. However, they present a lower ionic conductivity. Looking at the structure of the copolymers, it can be assumed that the ion motion between coordinating sites could be somewhat affected by the chlorine group of the PEO/EP polymer and that the PEO/EM/AGE presents a rigid chain structure and steric impediments.

Finally, the difference between PEG and PEO may be related to the low molecular weight of the former, 500 times lower than PEO. It has a hydroxyl group in both chain ends, whereas PEO has only one, the other end being a methoxy group (Figure 2). This means that the hydroxyl concentration is 1000 times larger in PEG than in PEO. This is relevant, since the electronegativity of the oxygen molecules that form the ending hydroxyl group in PEG (Figure 2) may be beneficial for the lithium ion conduction.

**Figure 10:**
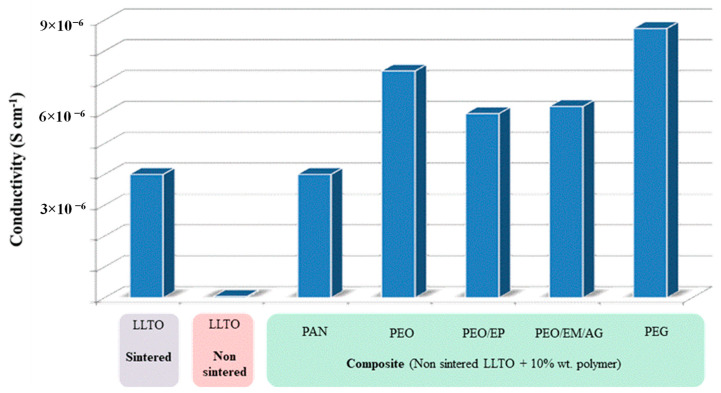
Total conductivity of non-sintered LLTO-polymer composites compared to both non-sintered and sintered LLTO.

Figure 10 summarizes the total ionic conductivity of composites using polymers (especially very low molecular weight PEG, which allows for the attainment of La_0.5_Li_0.5_TiO_3_-based thin films with reduced resistivity and low cost, overcoming the manufacturing constraints and brittleness of sintering. Note that, besides an improvement of two orders of magnitude with respect to non-sintered LLTO, the ionic conductivity of PEG-LLTO doubles that of sintered LLTO. The true mechanism for the increased conductivity of these composites may not be ascribed to any established mechanism of ion conduction through the polymer and would require further analysis beyond the scope of this work.

The conductivity of these samples still does not reach the value (10^−4^ S.cm^−1^) that allows them to be used as electrolytes in lithium batteries at room temperature. Further studies are being conducted to investigate the addition of lithium salts to these composites, with the final aim of testing these electrolytes in lithium ion batteries with conventional cathodes. On the other hand, the evaluation of other inorganic materials, such as LLZO, as well as the improvement of the processing conditions, would be of interest to evaluate the impact of this strategy [33].

## 4. Conclusions

Several La_0.5_Li_0.5_TiO_3_-polymer (low wt.%) composite solid electrolytes have been prepared using various polymers. In all cases, the polymer addition reduces the grain boundary resistivity compared to the sintered material and in two orders of magnitude in comparison to the non-sintered La_0.5_Li_0.5_TiO_3_. Therefore, a cheap and reliable procedure has been presented to prepare LLTO-based composites with similar conductivity to the corresponding sintered ceramic. A further reduction of grain boundary resistance by optimizing the polymeric processing may bring a practical composite to the field of lithium ion batteries.

Regarding particular polymers, impedance measurements reveal the highest ionic conductivity for the composite with 10 wt.% PEG non-sintered LLTO, 8.73 10^−6^ S cm^−1^ at room temperature, almost twice the conductivity of the inorganic sintered LLTO (4.10^−6^ S cm^−1^).

## Figures and Tables

**Table 1:** Principal advantages and disadvantages of the selected polymers used in this investigation.

Polymer	Advantages	Disadvantages
**PEO**	High ionic conductivity	High degree of crystallinity
**PEO copolymers**	Lower degree of crystallinity than PEO	Lower ionic conductivity than PEO
**PEG**	Higher ionic conductivity than PEO	Smaller polymer chains (lower Mw)
**PAN**	Good Li^+^ transport properties	Interface passivates the lithium anode

**Table 2:** Bulk and grain boundary resistivity and conductivity for sintered and non-sintered La_0.5_Li_0.5_TiO_3_.

		Sintered	Non-Sintered
Bulk resistivity (Ω cm)	R_1_	1.0 × 10^3^	1.0 × 10^3^
Grain boundary resistivity (Ω cm)	R_2_	2.5 × 10^5^	2.4 × 10^7^
Bulk conductivity (S cm^−1^)	1/R_1_	1.0 × 10^−3^	1.0 × 10^−3^
Grain boundary conductivity (S cm^−1^)	1/R_2_	4.0 × 10^−6^	4.2 × 10^−8^
Total conductivity (S cm^−1^)	1/(R_1_ + R_2_)	4.0 × 10^−6^	4.2 × 10^−8^
Error	χ^2^	0.008	0.009

**Table 3:** The total conductivity for La_0.5_Li_0.5_TiO_3_ and 10% polymer-composite samples obtained from bulk and grain boundary resistivities at room temperature.

	Total Conductivity (S cm^−1^)	Bulk Resistivity R_1_ (Ω cm)	Grain Boundary Resistivity R_2_ (Ω cm)
**PEO**	7.35 × 10^−6^	9.52 × 10^2^	1.42 × 10^5^
**PEG**	8.73 × 10^−6^	9.09 × 10^2^	1.12 × 10^5^
**PAN**	4.00 × 10^−6^	1.00 × 10^3^	2.52 × 10^5^
**PEO/EP**	5.97 × 10^−6^	9.43 × 10^2^	1.71 × 10^5^
**PEO/EM/AGE**	6.20 × 10^−6^	1.01 × 10^3^	1.63 × 10^5^

## Data Availability

The data of this study are available on request from the corresponding author.
